# Tissue-Specific Sample Dilution: An Important Parameter to Optimise Prior to Untargeted LC-MS Metabolomics

**DOI:** 10.3390/metabo9070124

**Published:** 2019-06-27

**Authors:** Zhanxuan E. Wu, Marlena C. Kruger, Garth J.S. Cooper, Sally D. Poppitt, Karl Fraser

**Affiliations:** 1 Food Nutrition & Health, Food and Bio-based Products, AgResearch Limited, Palmerston North 4442, New Zealand; 2 School of Food and Advanced Technology, Massey University, Palmerston North 4442, New Zealand; 3 High-Value Nutrition National Science Challenge, Auckland 1142, New Zealand; 4 School of Health Sciences, Massey University, Palmerston North 4442, New Zealand; 5 Riddet Institute, Massey University, Palmerston North 4442, New Zealand; 6 Centre for Advanced Discovery and Experimental Therapeutics, Division of Cardiovascular Sciences, School of Medical Sciences, Faculty of Biology, Medicine and Health, University of Manchester, Manchester M13 9NT, UK; 7 Human Nutrition Unit, School of Biological Sciences and Department of Medicine, University of Auckland, Auckland 1010, New Zealand

**Keywords:** LC–MS untargeted metabolomics, lipidomics, tissue metabolite profiling, sample preparation for metabolomics

## Abstract

When developing a sample preparation protocol for LC–MS untargeted metabolomics of a new sample matrix unfamiliar to the laboratory, selection of a suitable injection concentration is rarely described. Here we developed a simple workflow to address this issue prior to untargeted LC–MS metabolomics using pig adipose tissue and liver tissue. Bi-phasic extraction was performed to enable simultaneous optimisation of parameters for analysis of both lipids and polar extracts. A series of diluted pooled samples were analysed by LC–MS and used to evaluate signal linearity. Suitable injected concentrations were determined based on both the number of reproducible features and linear features. With our laboratory settings, the optimum concentrations of tissue mass to reconstitution solvent of liver and adipose tissue lipid fractions were found to be 125 mg/mL and 7.81 mg/mL respectively, producing 2811 (ESI+) and 4326 (ESI−) linear features from liver, 698 (ESI+) and 498 (ESI−) linear features from adipose tissue. For analysis of the polar fraction of both tissues, 250 mg/mL was suitable, producing 403 (ESI+) and 235 (ESI−) linear features from liver, 114 (ESI+) and 108 (ESI−) linear features from adipose tissue. Incorrect reconstitution volumes resulted in either severe overloading or poor linearity in our lipid data, while too dilute polar fractions resulted in a low number of reproducible features (<50) compared to hundreds of reproducible features from the optimum concentration used. Our study highlights on multiple matrices and multiple extract and chromatography types, the critical importance of determining a suitable injected concentration prior to untargeted LC–MS metabolomics, with the described workflow applicable to any matrix and LC–MS system.

## 1. Introduction

Untargeted metabolomics is becoming more widespread as a powerful tool for biomarker discovery and determination of metabolic changes associated with exposure, diet, and disease at a system level [1,2,3,4,5]. This involves measuring as many metabolites as possible, both the known and unknown molecules present in a biological sample, followed by data pre-processing to extract chemometric information and relative intensities of features from the spectral data, and subsequent analysis with multivariate/univariate statistical methods to identify discriminant features between groups of interest [6]. To date, liquid chromatography–mass spectrometry (LC–MS) is the preferred technique for untargeted metabolomics, enabling the most comprehensive metabolite coverage due to the compatibility of most metabolites with LC along with the sensitivity and selectivity of MS [7,8,9]. Although validated extraction methods suitable for various analytical platforms, tissue types and biofluids are available [10,11,12,13,14,15], careful selection of a suitable injected concentration (or sample loading amount) for each particular LC-MS system are sometimes overlooked or less considered when developing a sample preparation protocol. Injected concentration can be associated with overloading, signal saturation or features falling below detection limit and, hence, is a factor that can affect data quality and reproducibility [16]. A robust metabolomics method requires not just that a maximum number of metabolites is detected, but also that they fall within the linear dynamic range of the method to allow direct comparison of metabolites between samples [6]. Not accounting for nonlinearity of the measurements will severely affect the biological interpretation of the results.

With recent technological advances, a diverse range of LC–MS systems are available for untargeted metabolomics, spanning from the already commonly used systems such as high- or ultra-performance LC systems coupled to (Q-)TOF, orbitrap or triple quadrupole MS instruments [17], to more recently emerging analytical systems such as chemical isotope labelling (CIL)-nanoLC–MS and nanoLC-nanoelectrospray-MS [18,19]. The performance of different vendors LC–MS instruments systems can vary largely through factors such as ion source ionisation efficiency, ion extraction and focusing, and linear dynamic range of the MS detection system, as well as external variables such as the loading capacity of different LC separation columns. In a recent study by Cajka et al., the optimum loading amount of plasma extracts for lipidomics analysis was found to be instrument-dependent, and the author highlighted that avoiding ion saturation is the key to harmonise results across different laboratories [20]. The presence of sample-specific matrix effects could add further complexity with regards to ionisation mechanisms and interfering molecules impacting signal responses of individual metabolic features in a less predictable way [21]. Moreover, metabolite composition and abundance can vary widely for different sample types, hence, optimum injected concentration is also sample type-dependent and needs to be individually adjusted for.

In an untargeted metabolomics study, it is extremely difficult to use standard compounds to monitor signal linearity for every single feature due to the presence of unknowns [22]. Nonetheless, evaluation of the linear dynamic range can been performed using a serial diluted pooled quality control (QC) sample [20]. A strategy that included a serial diluted pooled QC in an analytical run sequence along with the analysis of experimental samples, followed by removal of features that did not follow proper linear trends during data pre-processing step, has been previously proposed to improve data robustness and quality [23,24]. Despite its usefulness in discriminating true biological signals from non-biological origin features, this strategy could also discard biological features that are already at a suboptimal concentration in the samples due to incorrect sample reconstitution. Carefully adjusting the reconstitution volume and determining a suitable injected concentration prior to the analysis of real experimental samples will help to ensure the optimum number of reproducible and linear features in a single analytical run of experimental samples are measured.

The goal of this study was to develop a strategy for the determination of optimal injected concentrations for untargeted LC–MS metabolomics analysis of multiple tissue and extract types. The approach includes LC–MS analysis of serial diluted pooled extracts, chromatographic visualisation, reproducibility assessment and concentration-intensity correlation calculation, and should be carried out before analysing any experimental samples. Our study highlights that the reconstitution volume (thus sample concentration injected into the LC-MS) must be carefully adjusted to avoid overloading and signal saturation, and that injected concentration affects feature reproducibility and, hence, is a critical and independent factor to check to ensure accurate and reliable data. The usefulness of the developed strategy is not limited to the animal samples exemplified in the present study but can be applied to a variety of other sample types and LC–MS systems.

## 2. Materials and Methods

### 2.1. Animal Tissues for Sample Preparation Optimisation

Pig tissues were purchased from commercial butcheries (Palmerston North, New Zealand). The samples comprised a fresh section of subcutaneous adipose tissue from the loin area and liver.

### 2.2. Chemicals

All organic solvents for metabolite extraction, reconstitution and LC–MS analysis (chloroform, methanol, acetonitrile isopropanol and formic acid) were obtained from Thermo Fisher Scientific (Auckland, New Zealand) and were of LC–MS grade except chloroform, which was of analytical grade; Milli-Q^®^ ultrapure water was obtained from Merck Millipore (Bedford, MA, USA). Ammonium formate (Fluka™, HPLC grade) was obtained from Sigma-Aldrich (Auckland, New Zealand). Lipid internal standard 1-palmitoyl(D31)-2-oleoyl-sn-glycero-3-phosphoethanolamine (16:0 d_31_-18:1-PE) was purchased from Avanti^®^ (Avanti Polar Lipids, Alabaster, AL, USA).

### 2.3. Metabolite Extraction

The tissue extraction protocol has been previously described [25]. Each tissue type was divided into 20 samples weighing 50 mg each. To extract metabolites, 50 mg tissue (adipose tissue, liver) was homogenised in 800 µL pre-chilled (−20 °C) CHCl_3_:MeOH (50:50, *v*/*v*) with a 5 mm zirconium bead per plastic tube for 2 × 60 s at 30 Hz using a TissueLyser (Qiagen, Hilden, Germany), followed by addition of 400 µL water, vortex-mixing (2 × 15 s) and centrifugation (Eppendorf Centrifuge 5427 R, Germany). Centrifuge parameters were set at 11,000 rpm, 4 °C, 10 min. Two blank samples were prepared following exactly the same protocol except that there was no tissue present. For each tissue type, 200 µL of the lower organic layer from each sample was transferred into a glass tube, combined and gently mixed to generate an organic extract pool, which was subsequently aliquoted into 200 µL samples, evaporated to dryness under a stream of nitrogen and stored at −80 °C until analysis. Similarly, pooled polar extract was made by combining 200 µL aliquots of the upper aqueous layer from each sample from the same tissue type, mixing and then dividing the pooled extract into 200 µL aliquots, and again evaporating to dryness under a stream of nitrogen and stored at −80 °C until analysis.

### 2.4. Serial Dilution Experiment for Reconstitution Volume Determination

Dried organic extracts from 200 µL aliquot of the pooled sample for each tissue type were re-dissolved in different volumes (ranging from 100–6400 µL) of a modified Folch solution (CHCl_3_:MeOH:H_2_O, 66:33:1, *v*/*v*/*v*) containing pre-dissolved 0.01% 16:0 d_31_-18:1-PE internal standard [0.01% (%*w*/*v*)]. Dried aqueous extracts from 200 µL aliquot were re-dissolved in different volumes (ranged from 50 µL to 800 µL) of acetonitrile:H_2_O (50:50, *v*/*v*). The reconstitution solvent volumes and full range of tested injected concentration for the organic and aqueous extracts from each tissue type was summarised in Table 1. Note: Higher reconstitution volumes were utilised for adipose lipid extracts as a preliminary study utilising the same reconstitution volumes as liver produced severe overloading. Concentration of the extract injected was calculated as in the equation below:

$$Concentration\;\left(\mathrm{mg}/\mathrm{mL}\right)=\frac{\frac{50\;mg\;tissue}{Extraction\;solvent\;volume\;\left(\mathrm{mL}\right)\;†}\times 0.2\;\mathrm{mL}\;aliquot}{\frac{Reconstitution\;solvent\;volume\;\left(µL\right)}{1000}}$$

† Extraction solvent for polar metabolite = 0.8 mL; Extraction solvent for lipid = 0.4 mL

### 2.5. Ultra-Performance Liquid Chromatography (UPLC)-Mass Spectrometry Analysis of Lipids

LC–MS conditions were slightly modified from a previously described method by Samuelsson et al. [26]. Lipid analyses were performed using an Accela 1250 quaternary UHPLC system coupled to Q Exactive hybrid quadrupole-Orbitrap mass spectrometer (Thermo Fisher Scientific, Waltham, MA, USA). An Acquity CSH™ C18 column 1.7 µm, 2.1 mm × 100 mm (Waters, USA) was used for lipid separation with a column temperature of 65 °C and mobile phase flow rate at 600 µL/min. The mobile phases consisted of acetonitrile/H_2_O (60:40) with 10 mM ammonium formate and 0.1% formic acid (A), and isopropanol/acetonitrile (90:10) with 10 mM ammonium formate and 0.1% formic acid (B). Analytes were eluted from the column with the following gradient program: 15–30% B (0.0–2.0 min), 30–48% B (2.0–2.5 min), 48–82% B (2.5–11.0 min), 82–99% B (11.0–11.5 min), 99% B was maintained for 3.5 min followed by re-equilibration with 15% B for 5 min. Two microliter reconstituted samples were injected. Each sample was injected six times; three technical replicates with the mass spectrometer operating in positive and three technical replicates operating in negative ionisation mode with a heated electrospray ionisation source set to 370 °C. External mass calibration of the Orbitrap prior to sample analysis was performed by flow injection of the calibration mix solution according to the manufacturer’s instructions. High resolution data (resolution 70,000) was acquired by full scan from *m/z* 200–2000 with source voltage of 3500 V electrospray ionisation positive mode (ESI+) or −3600 V ESI negative mode (ESI−), capillary temperature of 275 °C, and sheath, auxiliary and sweep gas flow rates of 40, 10 and 5 arbitrary units, respectively.

### 2.6. Liquid Chromatography (LC)-Mass Spectrometry Analysis of Polar Metabolites

The LC–MS conditions used in this study were as previously described [27]. Briefly, polar metabolites were analysed with an Accela 1250 quaternary UHPLC pump coupled to an Exactive Orbitrap mass spectrometry (Thermo Fisher Scientific, Waltham, MA, USA). Chromatographic separation was carried out at 25 °C on a SeQuant^®^ ZIC^®^-pHILIC 5 µm, 2.1 mm × 100 mm column (Merck, Germany) with the following solvent system: A = 10 mM ammonium formate in water, B = 0.1% formic acid in acetonitrile. A gradient program was used at a flow rate of 250 µL/min: 3–3% A (0.0–1.0 min), 3–30% A (1.0–12.0 min), 30–90% A (12.0–14.5 min), 90% A was maintained for 3.5 min followed by re-equilibration with 3% A for 7 min. An injection volume of 2 µL was used and each sample was injected six times; three technical replicates with the mass spectrometer operating in positive and three technical replicates operating in negative electrospray ionisation mode. The electrospray probe was operated unheated at room temperature (20 °C) to avoid degradation of thermally labile compounds. External mass calibration of the Orbitrap prior to sample analysis was performed by flow injection of the calibration mix solution according to the manufacturer’s instruction. High resolution data (resolution 25,000) was acquired by full scan from *m*/*z* 55 to 1100 with source voltage of 4000 V for ESI+ and −4000 V for ESI−, capillary temperature of 325 °C, and sheath, auxiliary, and sweep gas flow rates of 40, 10, and five arbitrary units, respectively.

### 2.7. Data Processing

The acquired spectral data were converted with the ProteoWizard tool MSConvert (v 3.0.1818) to mzXML format and pre-processed with the XCMS package (v3.0.2) in the R environment (v3.2.2) [28], to extract chemometric information (metabolic features) and integrate the peak area (intensity) of the detected metabolic features. Solvent front (retention time < 2mins) was removed from analysis. Chromatographic visualisation was performed using Xcalibur™ Software (Thermo Fisher Scientific, USA). Total ion chromatograms (TIC) were visually examined to investigate the overall changes of total ion intensity as well as metabolite/lipid profiles. The extracted ion chromatograms (EIC) of peaks selected from highly apparent/abundant elution regions along with low abundant peaks from the baseline region were also assessed for Gaussian shape and visual trends of signal response relative to the injected concentration. XCMS parameters for peak detection in lipid and HILIC data are provided in Table S1. The pre-processed data was subjected to blank features filtering based on tstat and *p*-values (sample vs. blank tstat < 1 or those with tstat >1 but *p*-value ≥ 0.05) generated by the diffreport function from the XCMS package. Subsequent analyses were conducted on non-blank features and all results and discussion were based on non-blank features only, defined as having a sample vs. blank tstat > 1 and *p*-value < 0.05. The relative standard deviation (RSD) of each injected concentration for every feature was calculated based on the triplicate injections. Peak areas were log10 transformed and used to calculate the Pearson correlation coefficient (*r*) between intensity and concentration to provide an estimate of linearity. The mean intensity-concentration relationship cut-off value selected to represent good linearity was *r* > 0.95 for lipid features and *r* > 0.9 for HILIC features. Linearity calculations were repeated five times, firstly covering the full concentration range, and then excluding the lowest, two lowest, highest and two highest concentrations sequentially. The resulting features meeting the linearity requirements utilising the correlation and exclusion procedure described above were then gathered to yield the maximum number of linear features, thus determining the optimal concentration to be used for analysis.

## 3. Result and Discussion

### 3.1. Workflow Summary and General Considerations

A step of solvent evaporation and reconstitution has become commonly used in untargeted metabolomics, allowing for changes in injection solvent composition and the injected concentration of the sample extracts, ensuring optimal chromatography along with maximal metabolome coverage and/or minimal saturation is achieved [29]. It has been previously reported that optimum loading amount for plasma lipidomics analysis was instrument-dependent [20,30]. The present study highlights that the optimum injected concentration is also sample type-dependent and should, therefore, be adjusted individually for each tissue matrix and analytical stream. We developed a simple workflow for the determination of suitable injected concentrations for animal tissues metabolomics and lipidomics analyses, to maximise the number of features that fall within the linear range of analysis.

The developed workflow consisted of four steps: serial dilution and analysis of a pooled sample, brief visual chromatographic examination, data processing to summarise the number of features and their measured peak areas, followed by peak area response linearity assessment using calculated correlation coefficients. The selection of an initial concentration range to test began with the highest possible concentrations as it was likely to allow more low abundant metabolites to be detected [31]; however, this sample pre-concentration was also most likely to produce overloading and ion suppression and potentially introduce changes in the matrix [22]. To ensure injections causing excessive system overloading were quickly eliminated from the process visual chromatogram examination was initially carried out before entering the more time-consuming data pre-processing step. This step provided a quick view of the systematic effect of injected sample dilution on the acquired profile, peak shape, and linear trend. To perform this visualisation step, stacked or overlaid TIC and selected EIC of small, medium, and large peaks on a fixed intensity scale were examined. Peaks with height and area in the TIC and selected EICs increased as the concentration increased in response (e.g., peak height, ion abundance, peak area) to concentrations whilst maintaining Gaussian shapes without severe distortion (e.g., widening, shoulder peak split peak, flattened apex) were considered as acceptable and the tested concentration ranges were passed onto the next step for further data processing. If the visual inspection was not passed, the serial dilution experiment could be repeated with a higher or lower concentration range if there were no practical limitations, e.g., available sample amount. Once the visual chromatographic examination was satisfied, data processing was carried out to examine the effects of injected concentration on the number of detected features and their reproducibility. Correlation coefficients for the concentration-dependent response of every feature were calculated. This step covered the full range of testing concentrations as well as excluding one or two concentrations at either the higher or the lower end. When the highest (one or more) concentrations were excluded and the correlation calculations produced more linear features than that from the full concentration range, it was considered that there was a considerable number of chromatographically overloading features or features undergoing signal saturation or suppression. Likewise, excluding the lowest (few) concentrations produced more linear features, signifying that several features are under the detection limit when a low concentration is injected. This evaluation step informed how high or low the injected concentration may go without causing too much overloading/saturation or information loss. The general rule we followed to determine the suitable injected concentration for the analysis of our (or any) sample type was that the highest possible concentration within the concentration range that produced the maximum number of linear features should be selected.

### 3.2. Chromatographic Examination

**Figure 1:**
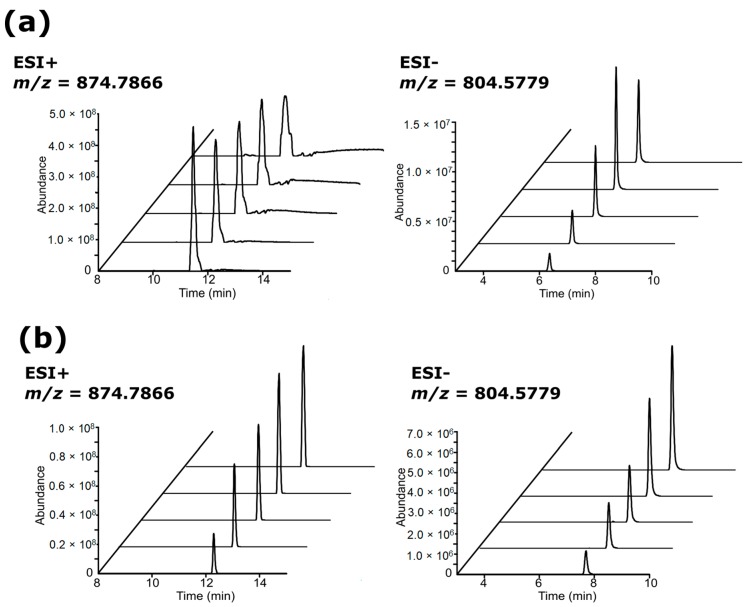
Extracted ion chromatogram (EIC) of selected features from adipose tissue lipid profile on a fixed scale of absolute intensity. EIC at (**a**) high injected concentrations (15.63–250 mg/mL) showed overloading in ESI+ and suppressed signal intensities in ESI−; data was from unpublished preliminary study and can be found in S1. This was improved by injecting at (**b**) lower concentration range (3.91–15.63 mg/mL) with higher dilution factors. The EIC along the *z*-axis starts from the lowest injected concentration at the front towards the highest concentration at the back.

**Figure 2:**
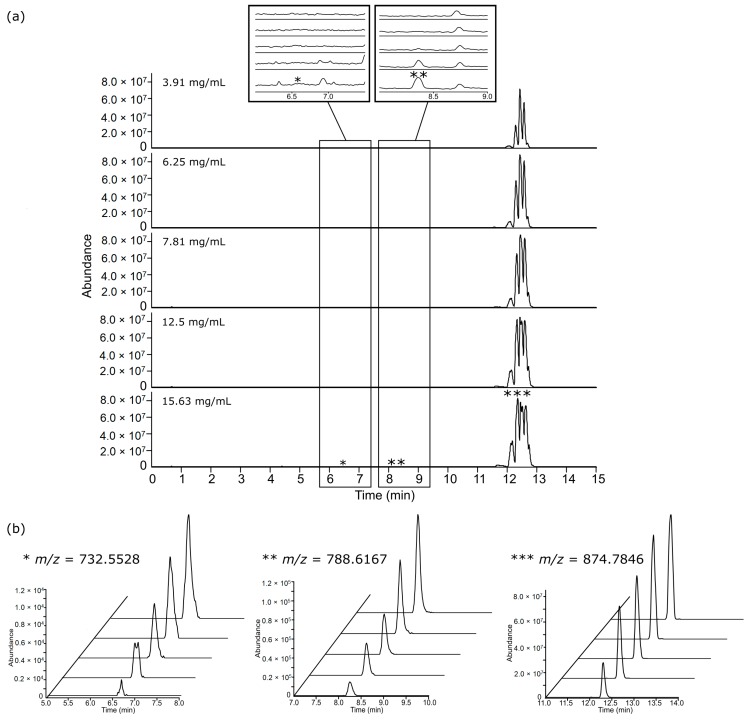
(**a**) Total ion chromatogram (TIC) of lipid extracts from 50 mg adipose tissue at low (3.91 mg/mL), intermediate (7.81 mg/mL) and high (15.63 mg/mL) injected concentration analysed by ESI+ and (**b**) examples for selected EIC of peaks representative of low, medium and high intensity features are indicated in the TIC with *, **, and ***, respectively, on a fixed scale of absolute intensity to evaluate peak shape and the concentration-dependent response. The EIC along the *z*-axis starts from the lowest injected concentration at the front towards the highest concentration at the back.

**Figure 3:**
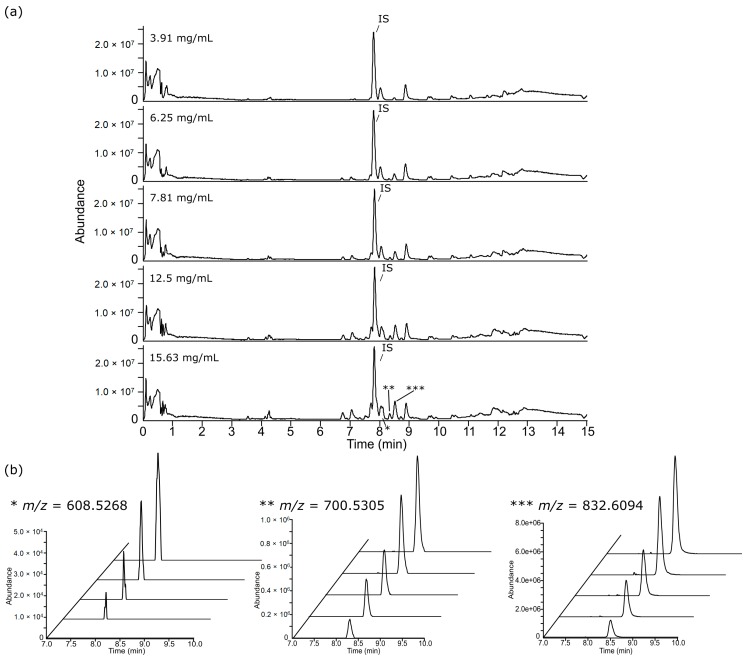
(**a**) Total ion chromatogram (TIC) of lipid extracts from 50mg adipose tissue at low (3.91 mg/mL), intermediate (7.81 mg/mL) and high (15.63 mg/mL) injected concentration analysed by ESI− and (b) examples for selected EIC of peaks representative of low, medium and high intensity features are indicated in the TIC with *, **, and ***, respectively, on a fixed scale of absolute intensity to evaluate peak shape and the concentration-dependent response. The EIC along the *z*-axis starts from the lowest injected concentration at the front towards the highest concentration at the back. IS: Internal standard peak.

Lipid extracts from liver and polar metabolite extracts from both adipose tissue and liver were injected at a concentration range between 15.63–250 mg/mL, whereas lipid extracts from adipose tissue were injected at a much lower concentration range between 3.91–15.63 mg/mL. This was due to severe chromatographic overloading of adipose lipid profile between 10–13 min analysed in ESI+, and supressed signal intensities between 5–9 min in ESI− that we observed in a preliminary study with a concentration range between 15.63–250 mg/mL (Figure S1). Incorrect reconstitution volumes, i.e., too concentrated lipid fractions from adipose tissue in this case, resulted in severe overloading and poor dilution responses, and this was improved by diluting the samples further (Figure 1). Examination of TICs and EICs of adipose tissue lipid profiles injected at 3.91–15.63 mg/mL showed overall good dilution responses and Gaussian peak shapes in ESI+ (Figure 2) and ESI− (Figure 3). Although trends of saturation at the higher concentrations were still observed and lower concentrations likely resulted in signal loss, these issues were further addressed and appraised using the subsequent step of linearity calculation and evaluation described below. It was also noted the importance of combining TICs and EICs for this step since relying solely on TICs examination could sometimes be misleading especially for regions with medium-to-low ionic intensities. For example, *m/z* 732.5528 from Figure 2 showed no apparent peak in the TIC, so performing EIC allowed testing of the dilution response for a concentration range of peaks. This highlighted that for the chromatographic examination step, it was important to examine not only regions of high intensity peaks, but also the baseline region. The three other tissue extracts (lipid for liver tissue, HILIC (polar metabolites) for liver tissue and adipose tissue) also passed both TICs and selected EICs evaluation step, showing overall good concentration-dependent responses and Gaussian peak shapes of the EICs (Figures S2–S4) and, thus, were passed through to the XCMS data processing step.

### 3.3. Feature Summary and Reproducibility

An increased number of features detected by XCMS was observed as injected concentration increased in both the lipid and HILIC analyses for ESI+ and ESI− of adipose tissue and liver tissue (Table 2). This was expected as lower injected concentrations tended to result in loss of signal of the lower abundant metabolites [32]. In the lipid data for both tissues, over 80% of the detected features in every tested concentration in both ionisation modes exhibited good reproducibility (relative standard deviation (RSD) < 30%), indicating that increasing the injected concentration did not impair reproducibility under conditions applied in this study.

Similarly, over 80% of the detected features in every tested concentration of both tissues analysed by HILIC ESI+ exhibited good reproducibility (RSD < 30%), whereas HILIC ESI− analysis produced less features as well as a generally larger RSD, especially at lower injected concentrations (≤31.25 mg/mL). Despite a generally good RSD analysed by HILIC ESI+, the total number of features and percentage of reproducible features were much lower at low concentrations (≤31.25 mg/mL). Our data showed that when injected concentrations were less than 31.25 mg/mL, a large portion of polar metabolic features from both adipose and liver tissues were not stably and reproducibly measured, and thus should be avoided. This highlighted that the injected concentration considerably impacted both the number of detected features and features reproducibility, especially with the HPLC HILIC analysis system, and therefore should be taken into consideration when selecting for a suitable injection concentration for LC–MS analysis.

### 3.4. Feature Linearity Testing for Selection of Suitable Injection Concentration

**Figure 4:**
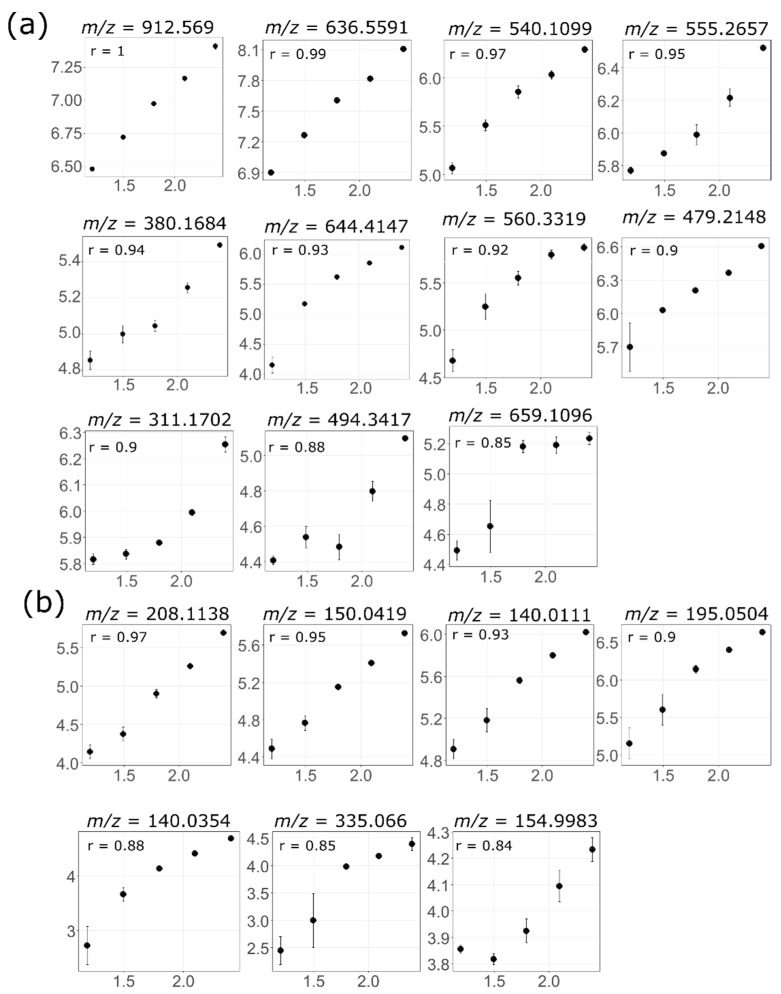
Examples for correlation plots of features in liver lipid ESI− (**a**) and liver HILIC ESI− (**b**), highlighting a linear-to-non-linear transformation from the above to the below of the cut-off r value (0.95 for lipid and 0.9 for HILIC).

Linearity assessment was performed on all features present in the highest injection concentration in each dataset, including those that might fall outside the detection limit when injected at a lower concentration. After careful assessment of the linear relationship between concentration and peak area response, a correlation coefficient (*r*) cut-off value of 0.95 for the lipid and 0.9 for the HPLC HILIC analysis was considered optimal for the two analytical datasets. The difference in cut-off value for lipid and HILIC analyses was due to an overall different data reproducibility by the two analytical platforms. RSDs were better for the UPLC lipid analysis than the HPLC HILIC analysis potentially due to higher signal-to-noise ratios and improved peak integration of the UPLC peaks [33,34], with the reproducibility of the peak measurement likely impacting the correlation coefficient. For example, the highest *r* value in the ESI− analysis of liver lipid and HILIC were 1 and 0.97, respectively, despite they were graphically similar in the degree of linearity (Figure 4). Figure 4 showed how progressive drops in *r* value corresponded to changes in the linear relationship between concentration and ion intensity on a correlation plot, and provides (some visual) references as to how an optimal cut-off value for linearity assessment was determined. As expected, a clear linear relationship was observed at the highest r value to start with. At the first few units drop in the r value, e.g., from 1–0.96 in lipid and 0.97–0.93 in HILIC analyses, the linear relationship was preserved. As *r* value dropped further, the linear relationship started to slightly distort or curve. A cut-off value was set at the margin of the well-preserved linearity and where subtle curvature may occur. In the following context, features that passed the cut-off value will be referred as linear features and the rest as non-linear features.

**Figure 5:**
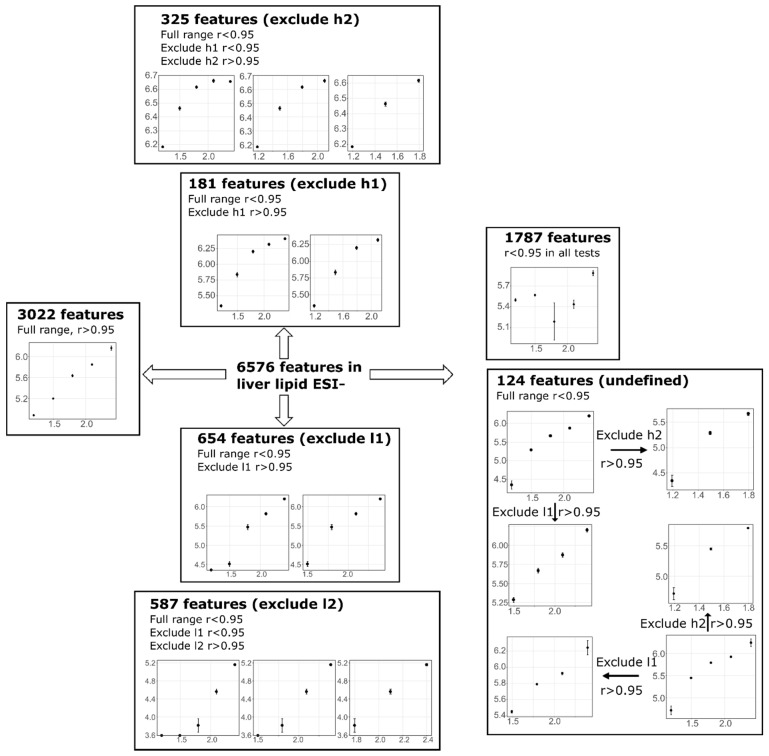
Features assigned to different category using liver lipid ESI− as an example. Excludedh1, h2, l1, l2 means excluding the highest one, highest two, lowest one, or lowest two concentrations. Excluding these concentrations improved r values of non-linear features to above the cut-off threshold respectively.

Non-linear features were mainly due to: 1. Large RSD in one or more concentrations at either the higher or lower concentration end; 2. Near plateau at either the higher or lower concentration range; and 3. Processing artefacts, matrix effect and substances from non-biological origin. Non-linear features due to the last reason above would have random trends or an inverse correlation for the signal-to-injection concentration response [35]. Whilst processing artefacts are inevitable, the number of features attributed to this category should be similar for the same tissue extract type across the five tested concentrations because the extraction protocol, instrument settings, and XCMS data pre-processing settings were kept identical. Non-linear features due to the first two reasons above would still produce a concentration-dependent trend and removal of the distortion region was expected to resume linearity. As such, the correlation coefficient was recalculated with the exclusion of one or two concentrations at either end of the injected concentration range. Non-linear features that became linear after exclusion of one or two concentrations would be assigned to one of the “exclude” categories; for example, if excluding the highest concentration made a non-linear feature become linear it was assigned/categorised as “exclude h1”. The number in each category was then compared to investigate how many non-linear features were due to higher end or lower end issues (e.g., overloading, below detection limit, high RSD, etc.). Figure 5 utilised liver lipid ESI− as an example to demonstrate this approach, and the result for all datasets were summarised in Table 3. Among the 6576 features detected, 3022 features exhibited linear response, and 3554 features were categorised as non-linear features when the dilution trend of full concentration range was used. Among these 3554 non-linear features, 654 features became linear after excluding the lowest concentration, and an additional 587 features became linear after excluding the lowest two concentrations, indicating a large number of features were problematic if injected at low concentration (<31.25 mg/mL). Possible reasons included samples were too dilute to be reproducibly measured or completely below the detection limit at these concentrations. On the other side, 181 and an additional 325 features became linear after excluding the highest and second highest concentrations respectively. The higher concentration end issue was likely to be associated with column overloading, saturation, signal suppression and/or samples became too concentrated to be reproducibly measured. A total of 1683 features remained non-linear even after excluding the higher or lower end datapoints. This can be attributed to several reasons, such as processing artefacts, signals from non-biological origin, incomplete removal of noise, or simply because the testing range did not cover the linear range of the feature. There was also a small amount of features falling in a category called “undefined” and only lipid data in the present study appeared to show this phenomenon. These features were non-linear in the full range but became linear both after excluding higher end and low end, primarily due to dual end issues for which the exact reasons were difficult to identify and the responses were less predictable. As the number of features falling in this category was tolerable in all four lipid datasets (<10% of total features), they were treated as non-linear features, rather than being assigned to any of the “exclude” categories. Collectively, the interpretation of this step indicated that in liver lipid ESI−, it was favourable to inject higher than 31.25 mg/mL. Similarly, in liver lipid ESI+ excluding l1 and l2 increased the number of linear features by 422 and an additional 299 respectively, whereas excluding h1 or h2 only slightly increased the number of linear features by 20 and an additional 25, respectively, suggesting an injected concentration higher than 31.25 mg/mL was favourable. Adipose lipid ESI− favoured an injected concentration higher than 3.91 mg/mL but ESI+ data indicated a considerable amount of overloading features if injected beyond 12.5 mg/mL, with excluded h2 increasing the number of linear features by 188. Therefore, it was desirable to also avoid an injected concentration higher than 12.5 mg/mL. All four HILIC datasets suggested an injected concentration no less than 31.25 mg/mL should be used for polar metabolites analysis.

Table 3 characterised feature linearity using the maximum number of detected features in each dataset, and informed about the concentrations that should be avoided. Yet it did not inform the number of linear features at each concentration that fell into the linear range. To this end, the total number of features at each concentration needed to be taken into account. Noted that the correlation coefficient was calculated based on all features detected in the highest concentration, this did not rule out the fact that some linear features might not meet the criteria of non-blank features at lower concentrations, i.e., although linear trend was observed, the signal-to-noise ratio, or integrated peak area, in the low concentration samples were not significantly different from blank samples. To calculate the number of linear features at each concentration, the data was first filtered based on the number of non-blank features at each concentration, followed by summing up/gathering the number of linear features at that concentration. The number of features that fell into the linear range at each concentration was summarised in Table 4. Again with liver lipid ESI− as an example, 1339 non-blank features detected in liver lipid ESI+ at the lowest concentration (15.63 mg/mL), among which 1285 were linear based on the full range calculation, 13 were linear in exclude h1, 16 were linear in exclude h2 and thus a total 1312 linear features out of the 1339 detected non-blank features were detected in the 15.63 mg/mL concentration sample. Linear features from exclude l1 and exclude l2 (i.e., non-linear became linear after excluding the lowest or lowest two concentrations) would not be counted as a linear feature for this concentration as they fell outside of the linear range when injected at 15.63 mg/mL. With this approach, it was concluded that an injected concentration of 7.81 mg/mL was suitable for lipid analysis of adipose tissue, yielding 698 (ESI+) and 498 (ESI−) linear features. An injected concentration of 125 mg/mL was suitable for lipid analysis of the liver, yielding 2811 (ESI+) and 4326 (ESI−) linear features, whereas in HPLC HILIC analysis it was optimal to inject at 250 mg/mL for both tissue types to maximise the number of linear features yield (Table 4).

### 3.5. Strength and Limitation of This Study

This study addressed an often-overlooked topic on the choice of suitable injected concentration for analysing samples in an untargeted metabolomics study. Reconstitution has been a way to alter sample concentration to suit the analytical platform and condition. The metabolite profile of different sample types can be very different in abundance of particular lipid species or metabolites due to the different biological processes and functions they carry out [36]. For example, lipid concentrations and compositions vary considerably between different tissues, such as adipose compared to muscle tissue. Even with the same extraction protocol and analytical platform, the injected concentration between differing tissues was an important parameter to check for as it affected both the profile and the amount of reliably measured features. Due to the different instrument sensitivities and column loading capacities equipped in different laboratories, in combination with the very different nature of the sample types of interest, the so-called suitable concentration could vary considerably. Few studies have detailed the decision on the suitable injected concentration when performing untargeted metabolomics, potentially for the reason that this decision could be subjective. This study attempted to demonstrate a workflow to this end. We have provided some references and guidelines as to how injected concentration could impact number of detected features, reproducible features and non-linear features. We have also provided examples to demonstrate potential detrimental consequences of not carefully checking the tissue-specific sample dilution prior to the analysis of real samples, hence highlighting the importance of this step.

Signal response to sample dilution can be used to evaluate signal linearity in untargeted metabolomics [24]. The use of serial diluted samples along with real samples at analytical and post-analytical stages to remove non-linearly scaled features to improve data quality has been previously reported [23,35,37]. Serial diluted samples can also be used to determine a suitable injection concentration for metabolomics analysis as a step of sample preparation protocol optimisation, which has yet to be described in existing literature. Here we are the first to describe a workflow with fine details for the determination of a suitable, sample type- and analytical stream-specific, injection concentration for metabolomics analysis. The described strategy was performed with a pooled sample to ensure the procedure was executed on a sample that will have the representative metabolite composition of the sample type and study condition. Whilst the study samples can be extracted, dried and briefly stored under optimal conditions [38], the pooled sample is used to run a serial dilution experiment; once the suitable injected concentration is determined, dried extracts from study samples can then be reconstituted and analysed at the optimum concentrations, although long-term storage of samples should be avoided as this could lead to sample degradation/poor resolubilisation and changes in profiles [39]. There are both pros and cons of this workflow. The process of data acquisition for the serial diluted pooled samples is identical to that for the study samples, hence, the signal responses to different injected concentration will reflect how study samples would behave. If a sample type is naturally abundant in certain class of metabolite, e.g., glycerolipids in adipose tissue [40], sample dilution can help to avoid column overloading, signal saturation and potentially ion suppression as well [41,42]. Sample pre-concentration, on the other hand, would allow more low abundant metabolites to be detected and reliably measured. On the down side, dilution of injected samples can cause signal loss of low abundant metabolites and hence a decrease of total detected features whereas sample preconcentration could cause overloading of abundant metabolites/lipid species. A higher dilution factor may also impair data reproducibility and increase RSDs as shown in this study. Given the wide concentration range of endogenous metabolites in samples and the goal of untargeted metabolomics is to reliably measure as many of them as possible it is, therefore, important to find the balance between data quality and metabolome coverage. This workflow, instead of looking at responses of individual lipid species or metabolites which would be not realistic in untargeted metabolomics, focused on the effect of injected concentration on metabolomic profile and feature properties on a global scale, and favoured the selection of concentration with the highest amount of linear features relative to the other tested concentrations. Another point worth noting is that increasing the number of reliably measured features did not necessarily mean an increased number of detected metabolites. Redundant features derived from isotopic masses, adduct formation and source-induced fragmentation could be presented without adding biological information to the data. Nonetheless, capturing extra chemometric information and maintaining data integrity might facilitate metabolite identification in the later stage of a metabolomics study. Lastly, this strategy has no means to optimise metabolome coverage or reproducibility from the aspect of extraction and reconstitution solvents as well as instrument settings, therefore, some knowledge on or a pre-optimised analytical workflow with regards to the choice of extraction and reconstitution solvents as well as the type of instrument and columns, elution programme, mobile phase, etc., should be established. There are many studies investigating extraction solvents and conditions for most commonly used sample types [13,14,17,38,43,44]. Nonetheless, our study highlights the critical importance of assessing the effect of injected sample concentration as an independent parameter on the acquired metabolomic profile, feature characterisation and linearity, and provides a feasible way to determine a suitable concentration prior to the analysis of real samples.

## 4. Conclusions

The present study utilised lipid and polar metabolite extracts (bi-phasic solvent extraction to generate extracts of polar metabolites and lipids) from two different tissues as examples to demonstrate a workflow for the selection of injected concentration for LC–MS analysis. We report under our laboratory and analysis conditions that an injected concentration at 125 mg/mL and 7.81 mg/mL is suitable for lipid analyses from liver and adipose tissue, respectively. Over 90% of the detected features were reproducible in each dataset, producing 2811 (ESI+) and 4326 (ESI−) linear features in liver and 698 (ESI+) and 498 (ESI−) linear features in adipose tissue. An injected concentration at 250 mg/mL was suitable for the analyses of polar extracts from both types of tissues. Over 85% of the detected features were reproducible in each dataset, producing 403 (ESI+) and 235 (ESI−) linear features in liver and 114 (ESI+) and 108 (ESI−) linear features in adipose tissue. We highlight that the injected concentration can be associated with overloading, saturation, or features under the detection limit, and affect data quality and reproducibility, as well as the number of detected and linear features. Therefore the injected concentration is a critical parameter to check for and should be carefully adjusted prior to the analysis of real samples. The optimum injected concentration could vary depending on various factors, such as extraction protocol, solvent compatibility, column type, instrument type and sample type, and we have demonstrated a general workflow to determine this important parameter. The developed workflow is also applicable to a variety of other sample types and LC–MS systems, and should be considered fundamental to LC–MS-based untargeted metabolomics analysis.

## Figures and Tables

**Table 1:** Reconstitution volume and concentrations of the analysed samples.

**Lipid**	**Reconstitution Volume (µL)**	**Concentration (mg/mL)**
Liver	100	250
	200	125
	400	62.5
	800	31.25
	1600	15.63
Adipose	1600	15.63
	2000	12.5
	3200	7.81
	4000	6.25
	6400	3.91
**Polar Metabolite**	**Reconstitution Volume (µL)**	**Concentration (mg/mL)**
Liver	50	250
	100	125
	200	62.5
	400	31.25
	800	15.63
Adipose	50	250
	100	125
	200	62.5
	400	31.25
	800	15.63

**Table 2:** Number of total detected non-blank features, number of non-blank reproducible features and percentage (%) reproducible features of total features at each concentration in lipid and polar (HILIC) fraction of adipose tissue and liver extracts.

-	Concentration (mg/mL)	ESI (+)			ESI (−)		
		#Non-Blank Features (tstat > 1, *p* < 0.05)	#Non-Blank Features with RSD < 30	%	#Non-Blank Features (tstat > 1, *p* < 0.05)	#Non-Blank Features With RSD < 30	%
Adipose lipids	3.91	856	814	95.1	441	420	95.2
	6.25	921	895	97.2	628	605	96.3
	7.81	939	914	97.3	751	710	94.5
	12.5	955	934	97.8	972	935	96.2
	15.63	985	942	95.6	1160	1127	97.2
Liver lipids	15.63	1339	1189	88.8	2984	2789	93.5
	31.25	1784	1510	84.6	4038	3820	94.6
	62.5	2634	2418	91.8	5122	4824	94.2
	125	3098	3047	98.4	5952	5659	95.1
	250	3210	3110	96.9	6576	6186	94.1
Adipose HILIC	15.63	20	17	85.0	30	29	96.7
	31.25	25	21	84.0	57	55	96.5
	62.5	77	72	93.5	72	62	86.1
	125	209	203	97.1	114	101	88.6
	250	239	217	90.8	176	154	87.5
Liver HILIC	15.63	53	47	88.7	39	11	28.2
	31.25	82	74	90.2	83	38	45.8
	62.5	435	413	94.9	236	221	93.6
	125	480	443	92.3	304	276	90.8
	250	553	496	89.7	380	349	91.8

**Table 3:** Number of features exhibiting linear trend from correlation analysis with full range of tested injected concentrations (*r* > 0.95 for lipids, *r* > 0.9 for HILIC) and number of additional features exhibiting linear trend after excluding the lowest concentration (exclude l1), excluding the lowest two concentrations (exclude l2), excluding the highest concentration (exclude h1) and excluding the highest two concentrations (exclude h2).

**Lipids**									
**Mode**	**Tissue**	**#Feature**	***r* > 0.95**	**Exclude l1**	**Exclude l2**	**Exclude h1**	**Exclude h2**	**Remain *r* < 0.95**	**Undefined**
ESI+	Adipose	985	428	23	5	56	188	235	50
	Liver	3210	2119	422	299	20	25	314	11
ESI-	Adipose	1160	304	121	42	25	47	576	45
	Liver	6576	3022	654	587	181	325	1683	124
**HILIC**									
**Mode**	**Tissue**	**#Feature**	***r* > 0.9**	**Exclude l1**	**Exclude l2**	**Exclude h1**	**Exclude h2**	**Remain *r* < 0.9**	**Undefined**
ESI+	Adipose	239	94	6	14	1	2	122	0
	Liver	553	349	16	38	9	7	134	0
ESI-	Adipose	176	70	26	12	0	1	67	0
	Liver	380	136	39	60	2	1	142	0

**Table 4:** The number of linear features that falls within the linear range and the number of linear features of the total detected non-blank features at each tested injected concentration in lipid and polar (HILIC) fractions of adipose tissue and liver extracts.

		Concentration	Non-Blank Features	*r* > 0.95 (Full)	*r* > 0.95 (Exclude l1)	*r* > 0.95 (Exclude l2)	*r* > 0.95 (Exclude h1)	*r* > 0.95 (Exclude h2)	Total Linear Features
Adipose lipids	ESI+	3.91	856	415	-	-	56	183	654
		6.25	921	426	22	-	56	187	691
		7.81	939	428	22	5	56	187	**698**
		12.5	955	428	22	5	56	-	511
		15.63	985	428	23	5	-	-	456
	ESI−	3.91	441	264	-	-	25	31	320
		6.25	628	290	95	-	25	35	445
		7.81	751	296	107	35	25	35	**498**
		12.5	972	302	119	41	25	-	487
		15.63	1160	304	121	42	-	-	467
Liver lipids	ESI+	15.63	1339	1285	-	-	13	14	1312
		31.25	1784	1570	145	-	15	16	1746
		62.5	2634	1939	344	198	18	21	2520
		125	3098	2083	414	295	19	-	2811
		250	3210	2119	422	299	-	-	**2840**
	ESI−	15.63	2984	2178	-	-	111	173	2462
		31.25	4038	2517	441	-	138	203	3299
		62.5	5122	2817	558	420	174	235	4204
		125	5952	2955	635	556	180	-	**4326**
		250	6576	3022	654	587	-	-	4263
Adipose HILIC	ESI+	15.63	20	4	-	-	0	1	5
		31.25	25	5	0	-	0	1	6
		62.5	77	29	0	7	1	1	38
		125	209	89	4	14	1	-	108
		250	239	94	6	14	-	-	**114**
	ESI−	15.63	30	27	-	-	0	0	27
		31.25	57	42	12	-	0	0	54
		62.5	72	49	17	3	0	0	69
		125	114	59	23	8	0	-	90
		250	176	70	26	12	-	-	**108**
Liver HILIC	ESI+	15.63	53	29	-	-	1	3	33
		31.25	82	42	2	-	3	5	52
		62.5	435	311	11	26	9	6	363
		125	480	331	14	30	9	-	384
		250	553	349	16	38	-	-	**403**
	ESI−	15.63	39	34	-	-	0	0	34
		31.25	83	60	5	-	2	0	67
		62.5	236	118	22	44	2	0	186
		125	304	126	35	51	2	-	214
		250	380	136	39	60	-	-	**235**

**Highest absolute number** is highlighted by bold and underlined.

## Supplementary Materials

The following are available online at https://www.mdpi.com/2218-1989/9/7/124/s1. Table S1: XCMS settings for peak detection in lipid data and HILIC data obtained from each ionisation mode. Figure S1: Total ion chromatogram (TIC) of lipid extracts from 50 mg adipose tissue at 15.63 mg/mL, 62.5 mg/mL and 250 mg/mL injected concentration and example for problematic extracted ion chromatogram (EIC) at this concentration range analysed by (a) ESI+ eluted between 8.5–14 min and (b) ESI− eluted between 5–9 min, on a fixed scale of absolute intensity. The EIC along the z-axis starts from the lowest injected concentration (15.63 mg/mL) at the front towards the highest concentration (250 mg/mL) at the back. The selected features for EIC examination were marked as * in the corresponding TIC. Figure S2: Total ion chromatogram (TIC) of lipid extracts from liver at low (15.63 mg/mL), intermediate (62.5 mg/mL) and high (250 mg/mL) injected concentration analysed by (a) ESI+ and (b) ESI−, and selected EIC of small (*), medium (**) and large (***) peaks representative of low, medium and high abundance features respectively on a fixed scale of absolute intensity to evaluate peak shape and the concentration-dependent response. The EIC along the z-axis starts from the lowest injected concentration at the front towards the highest concentration at the back. Figure S3: Total ion chromatogram (TIC) of polar extracts from adipose tissue at low (15.63 mg/mL), intermediate (62.5 mg/mL) and high (250 mg/mL) injected concentration analysed by (a) ESI+ and (b) ESI-, and selected EIC of small (*), medium (**) and large (***) peaks representative of low, medium and high abundance features respectively on a fixed scale of absolute intensity to evaluate peak shape and the concentration-dependent response. The EIC along the z-axis starts from the lowest injected concentration at the front towards the highest concentration at the back. Figure S4: Total ion chromatogram (TIC) of polar extracts from liver at low (15.63 mg/mL), intermediate (62.5 mg/mL) and high (250 mg/mL) injected concentration analysed by (a) ESI+ and (b) ESI-, and selected EIC of small (*), medium (**) and large (***) peaks representative of low, medium and high abundance features respectively on a fixed scale of absolute intensity to evaluate peak shape and the concentration-dependent response. The EIC along the z-axis starts from the lowest injected concentration at the front towards the highest concentration at the back.
