# Supplementary material for: Tissue-Specific Sample Dilution: An Important Parameter to Optimise Prior to Untargeted LC-MS Metabolomics
Source: Metabolites. 2019 Jun 27;9(7):124. doi: 10.3390/metabo9070124 (PMC6680868; doi:10.3390/metabo9070124)
Supplement: Supplementary file 1 [file metabolites-09-00124-s001.pdf]

Table S1: XCMS settings for peak detection in lipid data and HILIC data obtained from each ionisation mode

	Method	Ppm	Mzdiff	Peakwidth	Prefilter	Snthresh	Noise
**Lipid ESI+**	centwave	10	0.001	5, 30	6, 20000	20	10000
**Lipid ESI-**	centwave	10	0.001	5, 30	6, 20000	20	10000
**HILIC ESI+**	centwave	10	0.001	12, 48	3, 2000	20	1000
**HILIC ESI-**	centwave	10	0.001	12, 48	3, 2000	20	1000

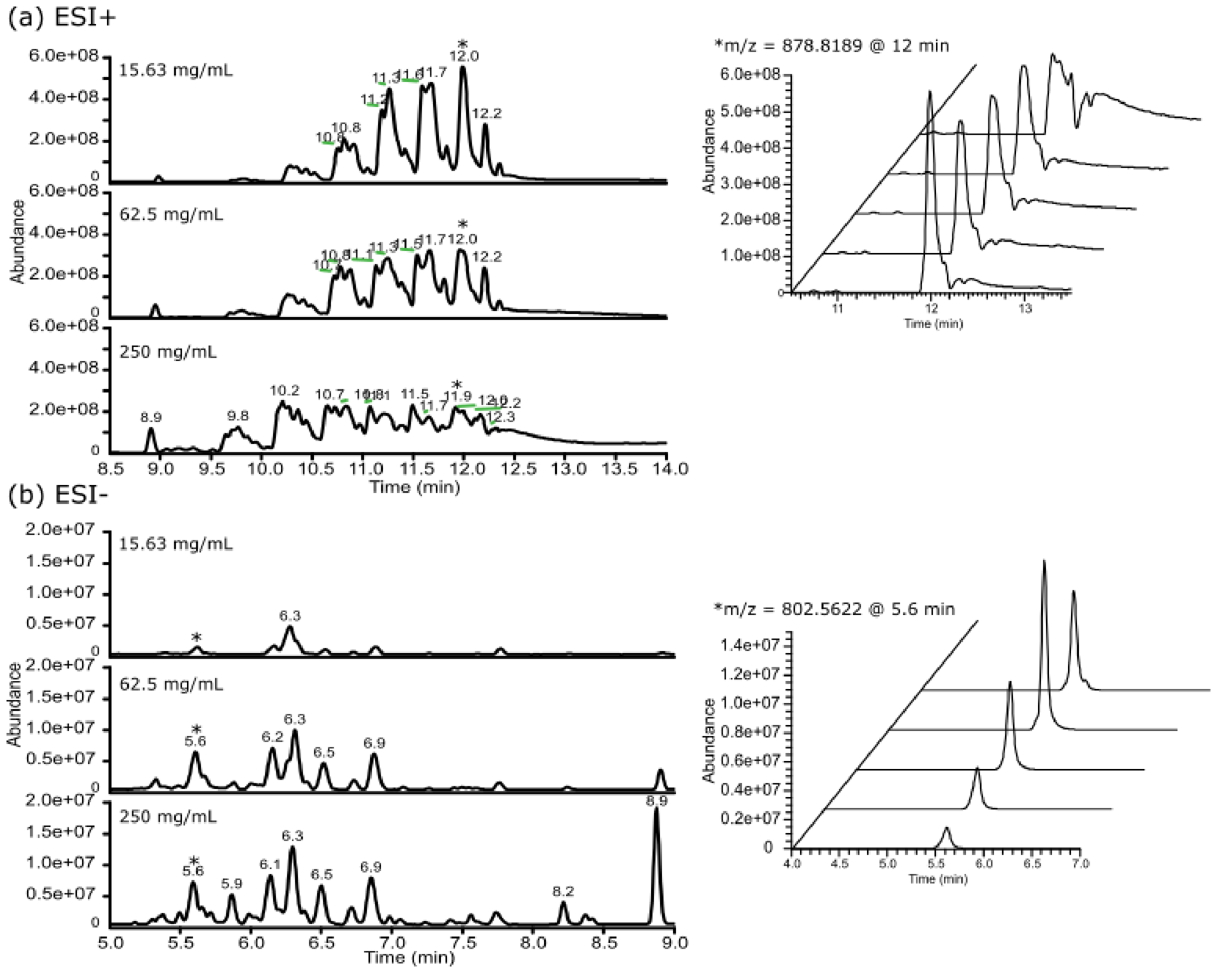

Figure S1: Total ion chromatogram (TIC) of lipid extracts from 50 mg adipose tissue at 15.63 mg/ml, 62.5 mg/ml and 250 mg/ml injected concentration and example for problematic extracted ion chromatogram (EIC) at this concentration range analysed by (a) ESI+ eluted between 8.5-14 min and (b) ESI- eluted between 5-9 min, on a fixed scale of absolute intensity. The EIC along the z-axis starts from the lowest injected concentration (15.63 mg/ml) at the front towards the highest concentration (250 mg/ml) at the back. The selected features for EIC examination were marked as * in the corresponding TIC.

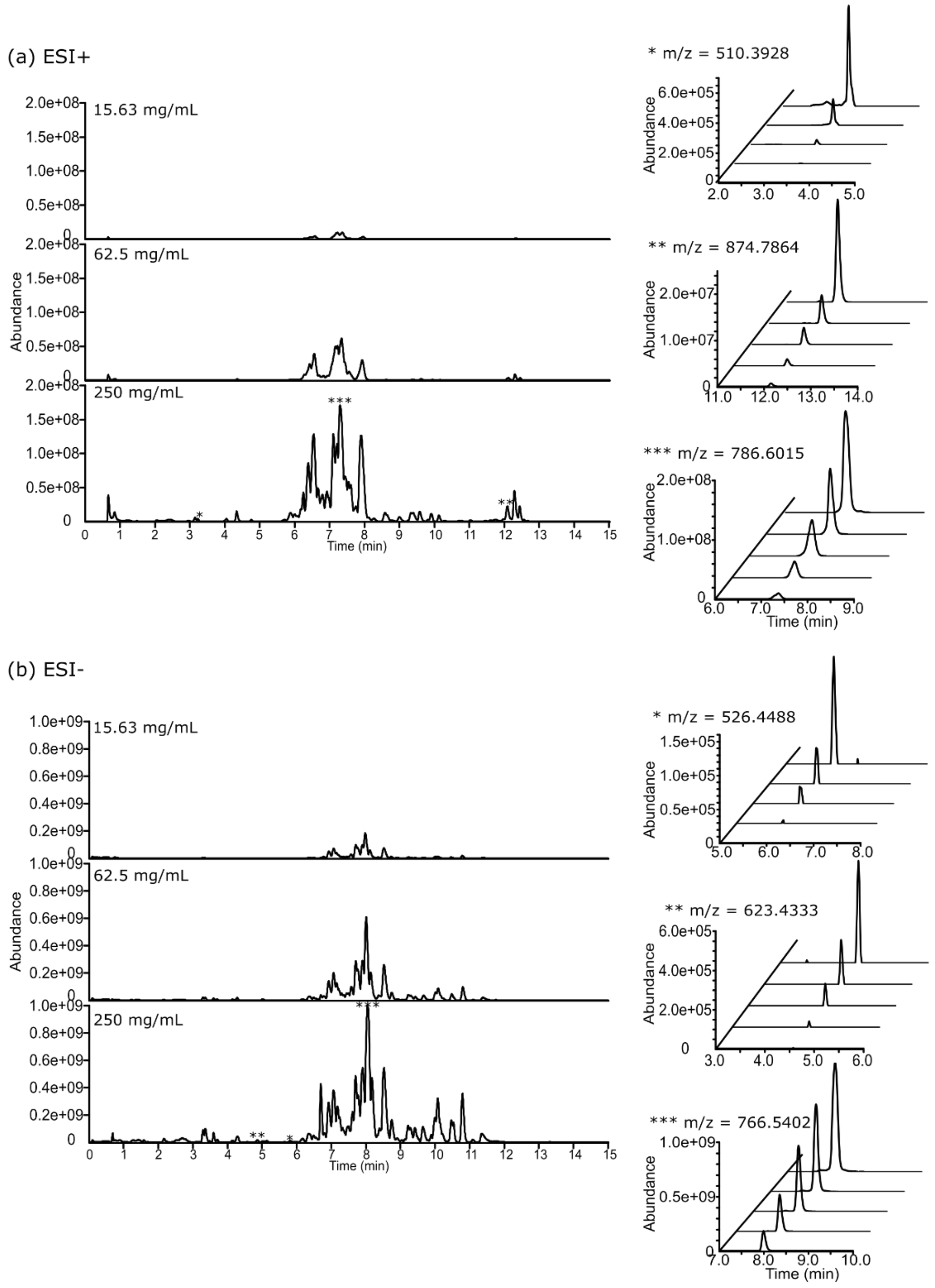

Figure S2: Total ion chromatogram (TIC) of lipid extracts from liver at low (15.63 mg/ml), intermediate (62.5 mg/ml) and high (250 mg/ml) injected concentration analysed by (a) ESI+ and (b) ESI-, and selected EIC of small (*), medium (**) and large (***) peaks representative of low, medium and high abundance features respectively on a fixed scale of absolute intensity to evaluate peak shape and the concentration-dependent response. The EIC along the z-axis starts from the lowest injected concentration at the front towards the highest concentration at the back.

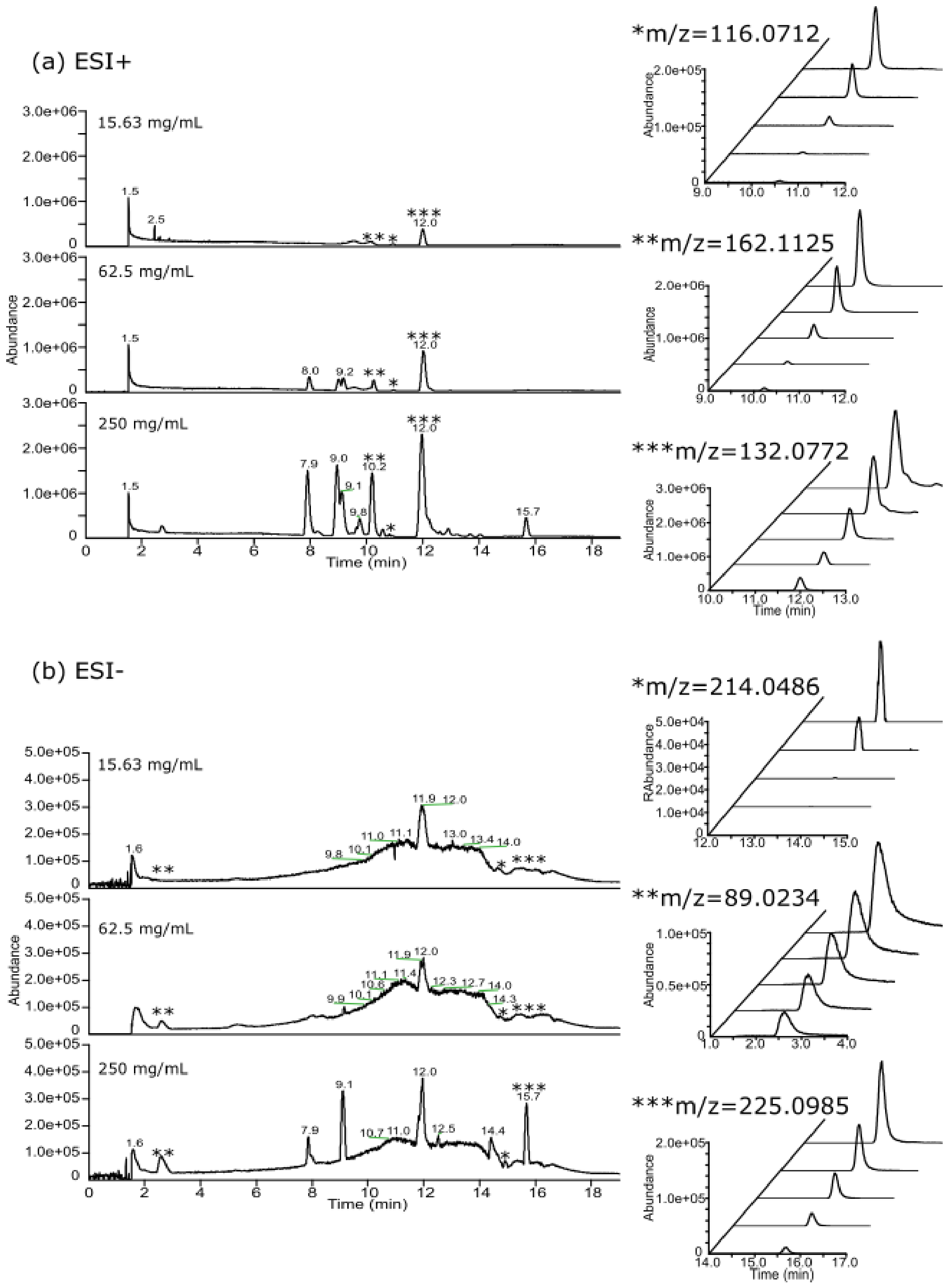

Figure S3: Total ion chromatogram (TIC) of polar extracts from adipose tissue at low (15.63 mg/ml), intermediate (62.5 mg/ml) and high (250 mg/ml) injected concentration analysed by (a) ESI+ and (b) ESI-, and selected EIC of small (*), medium (**) and large (***) peaks representative of low, medium and high abundance features respectively on a fixed scale of absolute intensity to evaluate peak shape and the concentration-dependent response. The EIC along the z-axis starts from the lowest injected concentration at the front towards the highest concentration at the back.

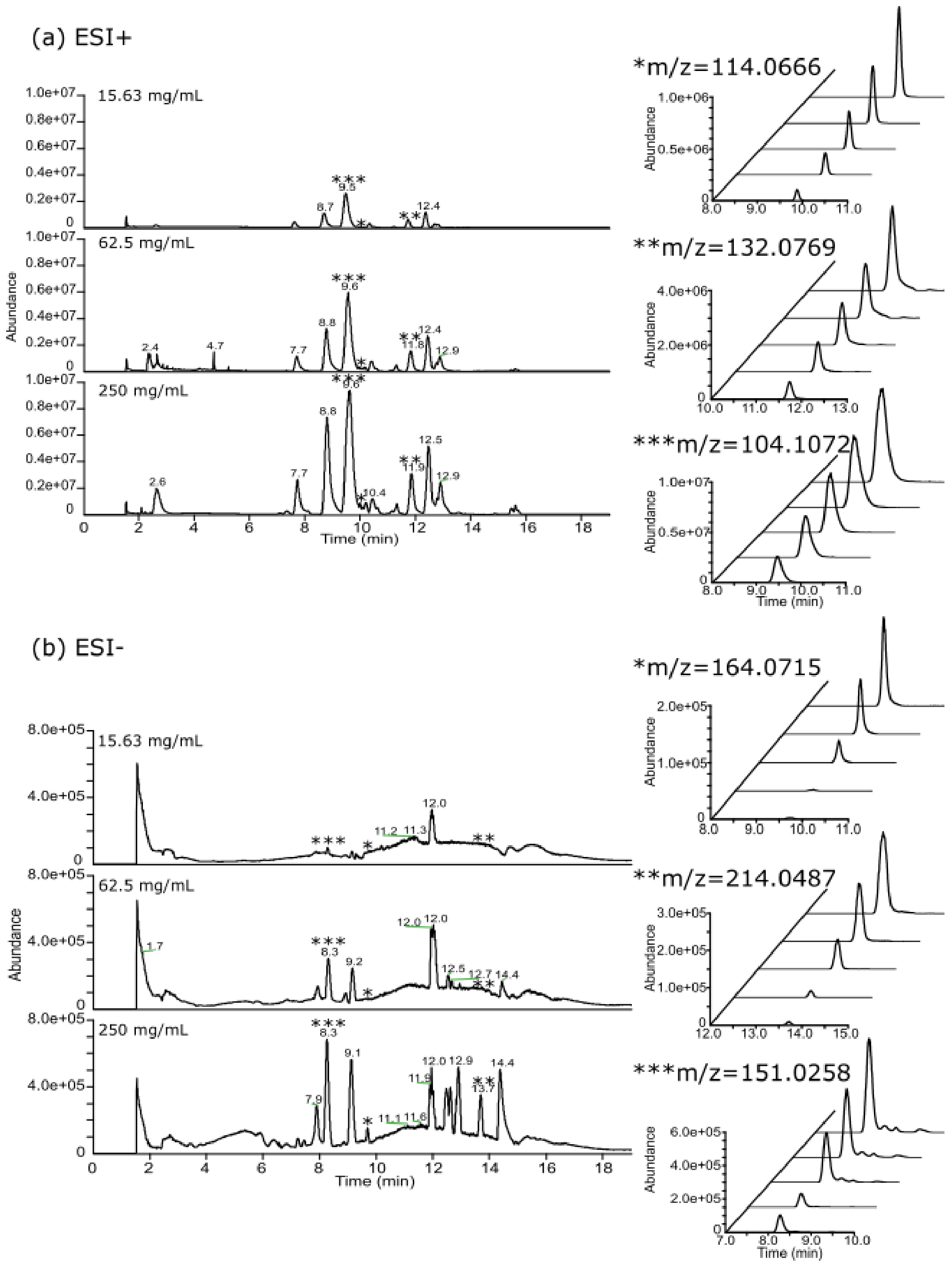

Figure S4: Total ion chromatogram (TIC) of polar extracts from liver at low (15.63 mg/ml), intermediate (62.5 mg/ml) and high (250 mg/ml) injected concentration analysed by (a) ESI+ and (b) ESI-, and selected EIC of small (*), medium (**) and large (***) peaks representative of low, medium and high abundance features respectively on a fixed scale of absolute intensity to evaluate peak shape and the concentration-dependent response. The EIC along the z-axis starts from the lowest injected concentration at the front towards the highest concentration at the back.
